# Colon cancer cell treatment with rose bengal generates a protective immune response via immunogenic cell death

**DOI:** 10.1038/cddis.2016.473

**Published:** 2017-02-02

**Authors:** Jianzhong Qin, Nicholas Kunda, Guilin Qiao, Jed F Calata, Krunal Pardiwala, Bellur S Prabhakar, Ajay V Maker

**Affiliations:** 1 Department of Surgery, Division of Surgical Oncology, University of Illinois at Chicago, Chicago, IL, USA; 2 Creticos Cancer Center, Advocate Illinois Masonic Medical Center, Chicago, IL, USA; 3 Department of Microbiology and Immunology, University of Illinois at Chicago, Chicago, IL, USA

## Abstract

Immunotherapeutic approaches to manage patients with advanced gastrointestinal malignancies are desired; however, mechanisms to incite tumor-specific immune responses remain to be elucidated. Rose bengal (RB) is toxic at low concentrations to malignant cells and may induce damage-associated molecular patterns; therefore, we investigated its potential as an immunomodulator in colon cancer. Murine and human colon cancer lines were treated with RB (10% in saline/PV-10) for cell cycle, cell death, and apoptosis assays. Damage-associated molecular patterns were assessed with western blot, ELISA, and flow cytometry. In an immunocompetent murine model of colon cancer, we demonstrate that tumors regress upon RB treatment, and that RB induces cell death in colon cancer cells through G2/M growth arrest and predominantly necrosis. RB-treated colon cancer cells expressed distinct hallmarks of immunogenic cell death (ICD), including enhanced expression of calreticulin and heat-shock protein 90 on the cell surface, a decrease in intracellular ATP, and the release of HMGB1. To confirm the ICD phenotype, we vaccinated immunocompetent animals with syngeneic colon cancer cells treated with RB. RB-treated tumors served as a vaccine against subsequent challenge with the same CT26 colon cancer tumor cells, and vaccination with *in vitro* RB-treated cells resulted in slower tumor growth following inoculation with colon cancer cells, but not with syngeneic non-CT26 cancer cells, suggesting a specific antitumor immune response. In conclusion, RB serves as an inducer of ICD that contributes to enhanced specific antitumor immunity in colorectal cancer.

The highest 5-year cancer-related mortality worldwide is secondary to solid organ gastrointestinal tumors, and the most common gastrointestinal tumor is colon cancer. The majority of patients with colon cancer will present with advanced disease, resulting in it being the second leading cause of cancer-related deaths in the United States.^1^ For most patients with metastatic colon cancer, palliative chemotherapy is the only present option; therefore, improved outcomes through new therapeutic strategies are desperately needed.

The presence of activated and proliferating T cells within primary colon tumors is associated with improved survival^2, 3^ and we have previously demonstrated an association between increased T-cell infiltrates and improved outcomes in patients with colon cancer metastases.^4, 5^ Thus, immunotherapy may have a viable role in managing patients with advanced gastrointestinal malignancies, including colon cancer, although mechanisms to incite tumor-specific immune responses remain to be elucidated for this disease.^6^

Rose bengal (RB), a synthetic dye used in the garment industry, was first patented in 1882 and has been used for many years in the medical field as a diagnostic of ocular pneumococcal infections, a measure of hepatic function, and as a stain for corneal ulceration.^7, 8, 9, 10, 11, 12^ RB 10% in saline, or PV-10, is not dependent on photostimulation for cytotoxic effects and is formulated for intralesional injection where it has been evaluated in phase I and II clinical trials for the treatment of in-transit metastatic melanoma. In these patients, direct injection of cutaneous deposits resulted in tumor destruction.^13, 14, 15^ Interestingly, occasional regression of non-injected bystander melanoma tumors occurred in these patients, raising the possibility that RB-induced cell death may generate an antitumor immune response.^14, 15, 16^ Therefore, we have evaluated the potential of RB-induced cell death to generate a tumor-specific immune response or to expose tumor antigens for T-cell presentation in various malignancies.^17^ Although we found that preclinical studies support that intralesional RB is capable of inducing cell death in multiple tumor cell lines without affecting normal dermal fibroblasts, the mechanism of generating an antitumor immune response remains to be elucidated. In these studies, it was found that RB entered cancer cells, but were excluded form normal cells, and that RB was not able to inhibit cell growth of normal human fibroblasts at concentrations that affected ovarian carcinoma, melanoma, and gastric cancer cells.^16, 18, 19, 20^

Immunogenic cell death (ICD) is heavily regulated and capable of activating an adaptive immune response against tumor-specific antigens.^21^ It is characterized by the release and/or increased expression of damage-associated molecular patterns (DAMPs), including ATP, HMGB1, heat-shock protein 90 (HSP90), and calreticulin (CRT), among other immunostimulatory molecules.^22, 23, 24, 25, 26, 27, 28, 29^ There is limited data evaluating the effect of RB on ICD in solid organ malignancies, including colon cancer, where there is established potential and a great need for immunotherapeutic strategies. The mechanism of RB-induced cell death and whether RB treatment may increase the immunogenicity of colon cancer cells is critical to determine if RB is to be used as an immunotherapeutic strategy in this disease.

## Results

### Intralesional treatment of established colon cancer cell tumors with RB induced significant tumor regressions *in vivo*

**Figure 1:**
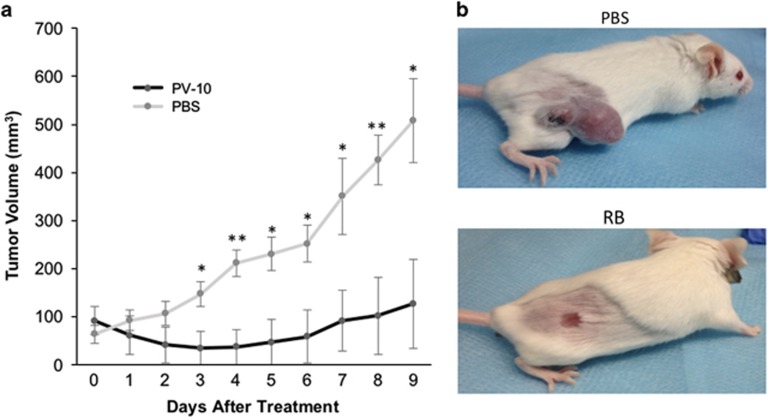
Effect of intralesional RB treatment on established tumors. (**a**) Subcutaneous tumors were established in the flanks of each mouse (*n*=6 per group, representative experiment of three). Animals were randomized and tumors were injected intralesionally with RB or PBS. Tumor growth was monitored daily. Animals were sacrificed when control animals reached humane end points (**b**). Representative animals demonstrate treatment response at day 9 to intralesional PBS (top panel) and RB (bottom panel). *P<0.05 and **P<0.01

To determine if RB treatment was a viable antitumor strategy for patients with colon cancer, it was critical to determine if intralesional RB had an antitumor effect on established colon cancer cell tumors. An immunocompetent Balb/C murine model using the syngeneic murine colon cancer cell CT26 was used.^30^ To characterize the effect of RB on established CT26 subcutaneous tumors, tumors were treated intralesionally with RB. Within one day of injection with RB, tumors decreased in volume and showed evidence of clinical ulceration. By 3 days, the difference in tumor volumes between the RB-treated and control groups was significant and persisted over the length of the experiment (*P*<0.05; Figure 1a). The effect was durable after a single injection on day 0, with some animals experiencing complete tumor regressions (Figure 1b).

### RB treatment induced potent colon cancer cell death *in vitro*

**Figure 2:**
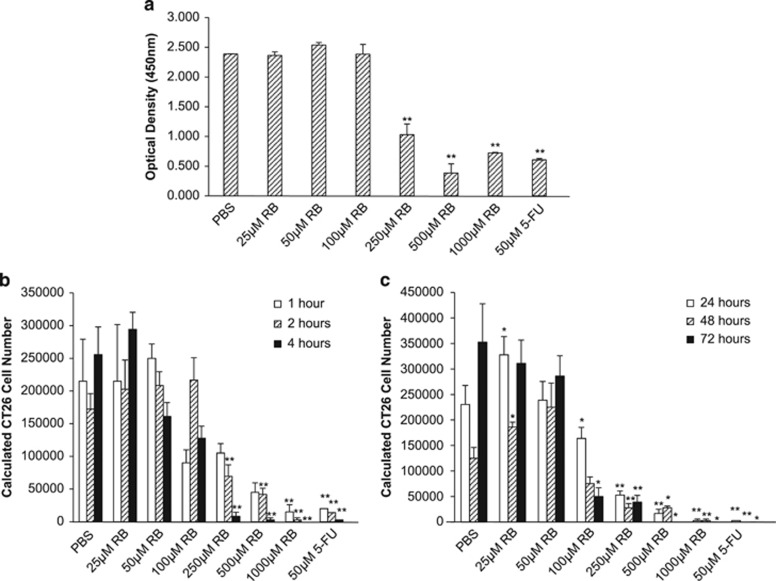
RB induces cell death in CT26 cells. (**a**) MTS assay performed after 24 h of RB treatment at various doses revealed cell death with increasing RB concentration. 5-FU, a known cytotoxic chemotherapeutic agent for colon cancer, was used as a positive control. Trypan blue exclusion at 1, 2, and 4 h (**b**) and 24, 48, and 72 h (**c**) demonstrated significant RB-induced cell death at similar doses (*P<0.05 and **P<0.01)

Once it was determined that RB treatment could impart clinical antitumor responses in established colon cancer cell tumors *in vivo*, we wished to further investigate the *in vitro* effects of titrated doses of RB on murine (CT26) and human colorectal cancer cells. Murine and human colorectal cells were treated with RB or 5-fluorouracil (5-FU) for 24 h. At RB concentrations >100 *μ*M, there was a significant decrease (*P*<0.01) in cell viability, as measured with an MTS assay, in the CT26 group when compared with the PBS-treated control group, which was similar to 5-FU chemotherapy-treated cells (Figure 2a). Similarly, in HT29 human colon cancer cells, a significant difference in viability was again seen upon exposure to RB, which increased from 250–1000 *μ*M (*P*<0.01) (Supplementary Figures 1C and D). Further, the MTS results were confirmed using quantitative observations in colon cancer cells treated with RB for 1, 2, 4, 24, 48, and 72 h. With increasing concentrations of RB, cell death increased as measured with Trypan blue exclusion. Significant cell death occurred after exposure to 100 *μ*M RB and increased to 1000 *μ*M RB at which point there was near-complete death of all cells (CT26 (Figures 2b and c) and HT29 (Supplementary Figure 1B)).

### RB induced G2/M growth arrest and predominantly necrosis in colon cancer cells

**Figure 3:**
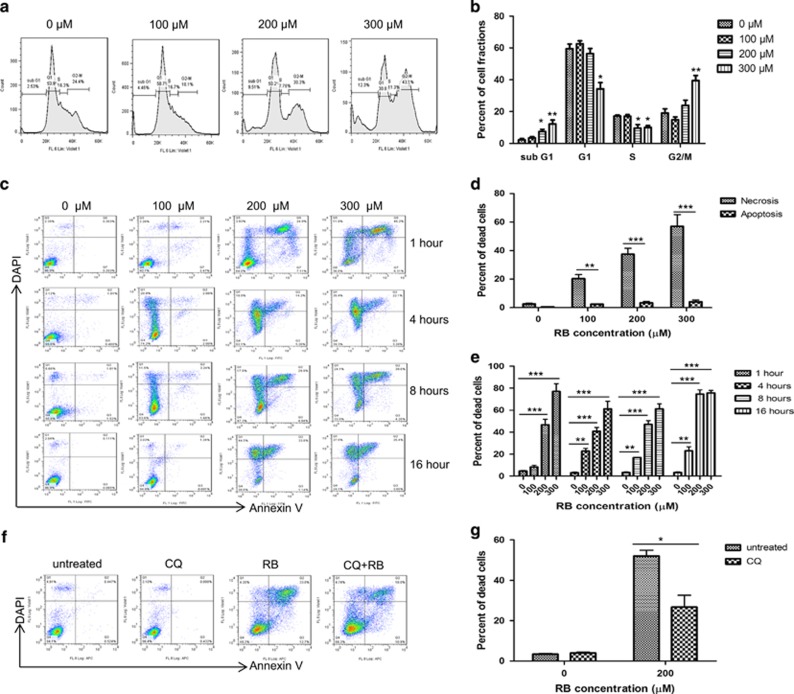
Mechanism of RB-induced cell death. (**a**,and **b**). Fluorometric-based cell cycle analysis was performed after 24 h RB treatment of CT26 colon cancer cells revealing G2/M growth arrest and DNA degradation when exposed to increasing doses of RB. Representative flow cytometry data are presented (**a**) and repeated in three separate experiments (**b**). (**c** and **d**). RB treatment induced cell death predominately by necrosis with increasing doses of RB. Representative flow cytometry data are presented (**c**) and repeated in three separate experiments comparing necrotic (DAPI+, DAPI+/Annexin V+) to apoptotic cell populations (DAPI−/Annexin V+) (**d**) (**e**). Time-course evaluation revealed that RB-induced cell death was initiated within 1 h, and by 4 h in all doses tested. (**f** and **g**) Cells were treated for 4 h with 200 *μ*M RB in the presence or absence of the autophagy inhibitor choloroquine (CQ) (*P<0.05, **P<0.01 and ***P<0.001)

After establishing that RB decreased cell viability of murine and human colorectal cells in a dose-dependent manner, we wished to determine the mechanism of cell death. Given that RB-induced cell death was evident upon exposure to concentrations >100 *μ*M, CT26 cells were treated with 100–300 *μ*M RB for 24 h for cell cycle analysis. At higher doses of RB, colon cancer cells displayed significant reduction of the cell fraction in the S phase and increased cell fractions in G2/M and sub-G1 phase, consistent with G2/M growth arrest (Figure 3a, representative flow cytometry analysis; Figure 3b, pooled data from three experiments). To examine the pattern of cell death, CT26 cells were treated with increasing concentrations of RB. RB induced cell death predominantly by cell necrosis, as evidenced by the significantly increased population of DAPI+ (4′, 6-diamidine-2′-phenylindole dihydrochloride) and DAPI/Annexin V-double-positive cells (Figure 3c). Minimal Annexin V-single positive cells at all examined time points support that necrosis was the predominant mechanism of cell death (Figures 3c and d).^31^ Cell death with RB was rapid with significant cell death observed with 200 and 300 *μ*M within an hour, and by 4 h at all doses tested (Figure 3e). To investigate RB-induced cell death in human colon cancer, HCT116, SW480 and HT29 cells were treated with the same doses of RB for 4 h (Supplementary Figure 2).

As premortem autophagy is required for ICD-associated secretion of ATP, CT26 cells were pretreated with chloroquine (autophagy inhibitor) and treated with RB (200 *μ*M) for 4 h. Chloroquine was found to significantly inhibit RB-induced cell death (Figures 3f and g).

### RB treatment induced DAMPs in colorectal cancer cells

That RB could induce *in vivo* and *in vitro* colon cancer cell death, and that the mechanism of cell death was primarily by rapid necrosis was determined; however, whether the induced death was immunogenic (ICD) remained to be evaluated. Therefore, we next sought to identify whether RB treatment of colon cancer cells induced the release of DAMPs.

#### Calreticulin

**Figure 4:**
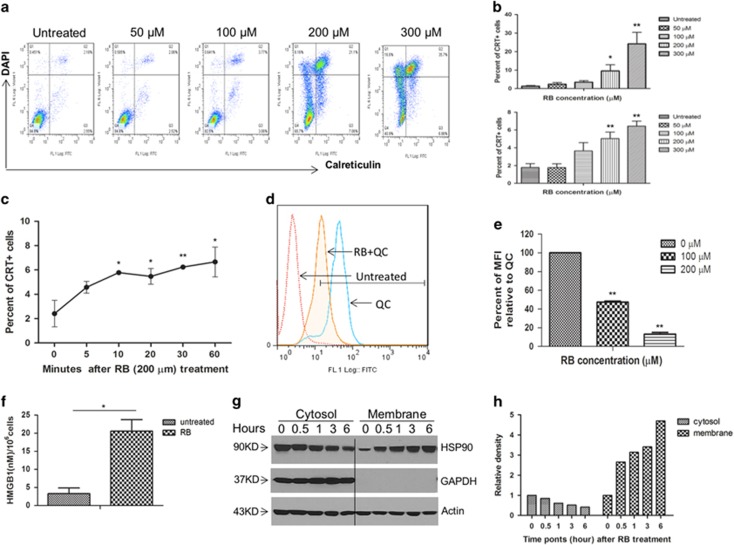
RB treatment induced the release and/or increased expression of DAMPs in colon cancer cells. (**a** and **b**) RB treatment of CT26 colon cancer cells induced increased surface expression of CRT in a dose-dependent manner (**a**). Increased expression was observed on both dead (**b**, upper panel) and live cells (**b**, lower panel) based on flow cytometry analysis after 1 h of treatment. (**a**) A representative example of three separate experiments summarized in (**b**). (**c**) CRT expression increased minutes after exposure to RB, reaching significance by 10 min. (**d** and **e**) ATP content in colon cancer cells was determined by cytofluorometric detection of intracellular ATP with quinacrine, expressed as the percentage of MFI relative to untreated cells. A representative histogram (**d**) and summary of three separate experiments (**e**) demonstrate a decrease in intracellular ATP levels upon treatment with RB. (**f**) HMGB1 secretion significantly increased as measured in culture supernatants after treatment with 200 *μ*M RB for 30 min. (**g** and **h**) RB treatment resulted in increased membrane expression and reduced cytosolic levels of HSP90 as the time of treatment increased from 30 min to 6 h as measured on western blot analysis (**g**) and after densitometric correction for housekeeping gene loading controls (**h**) (*P<0.05 and **P<0.01)

CRT expression was quantified, and the expression increased with RB treatment in a dose-dependent manner. This was evaluated on both live and dead cell populations after treatment (Figures 4a and b). Upregulated CRT expression was rapid in CT26 cells starting minutes after treatment and maintaining expression steadily for up to 1 h (Figure 4c). Upregulated CRT expression upon RB treatment was similar in multiple human colon cancer cell lines studied (Supplementary Figures 3A–C).

#### ATP

Mean fluorescence intensity (MFI) percentages of quinacrine were markedly decreased upon RB treatment relative to untreated CT26 cells in a dose-dependent manner, indicating that RB treatment results in diminished levels of intracellular ATP (Figures 4d and e). The results obtained by treating human colon cancer cell lines were similar to CT26 cells (Supplementary Figures 3D and E).

#### HMGB1

Passive cellular release of high-mobility group box 1 (HMGB1) enhances the function of dendritic cells and often occurs as cells are undergoing necrotic cell death. Extracellular HMGB1 protein level in the supernatants of cell culture significantly increased after treatment with RB (Figure 4f).

#### HSP90

To examine whether RB treatment induced HSP90 translocation to the cell surface, protein levels of HSP90 in cytosolic and cell membrane fractions were determined in RB-treated CT26 cells by western blotting. RB treatment led to reduced cytosolic levels of HSP90 and increased membrane expression as the time of treatment increased from 30 min to 6 h (Figures 4g and h). RB treatment of human colon cancer cell lines also increased the cell membrane expression of HSP90 in all tested cells (Supplementary Figure3G).

Collectively, these results indicated that treatment of colon cancer cells with RB induced DAMPs related to ICD.

### *In vivo* RB treatment generates a protective immune response

**Figure 5:**
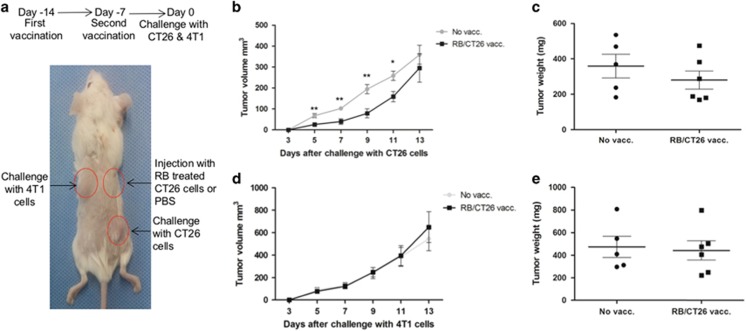
Vaccination with RB-treated cells induced early decreased tumor growth. (**a**) Injection locations and experimental timeline. (**b**) RB-treated CT26 tumor cell vaccination (vacc.) induced slower early challenge tumor growth in vaccinated mice. (**c**) CT26 challenge tumor weight trended to be decreased in vaccinated mice. (**d** and **e**) No difference was observed in tumor growth (**d**) or weight (**e**) of 4T1 mammary tumors in vaccinated compared with non-vaccinated animals (*P<0.05 and **P<0.01)

To determine if RB-treated CT26 cells stimulated a specific antitumor immune response *in vivo*, Balb/c mice were vaccinated with a subdermal injection of *in vitro* RB-treated CT26 cells on days −14 and −7 before rechallenge with untreated CT26 cells and syngeneic 4T1 breast cancer cells on day 0 (Figure 5a). In the 2-week period after tumor challenge, CT26 tumor growth was significantly reduced at early time points in vaccinated mice compared with the control group (Figure 5b). Colon cancer tumor weights from vaccinated mice trended to be less than sham-vaccinated animals (Figure 5c). In contrast, the growth curve of syngeneic non-CT26 4T1 control mammary cell tumors at the same time points were almost identical between vaccination and control groups, as were tumor weights (Figures 5d and e).

**Figure 6:**
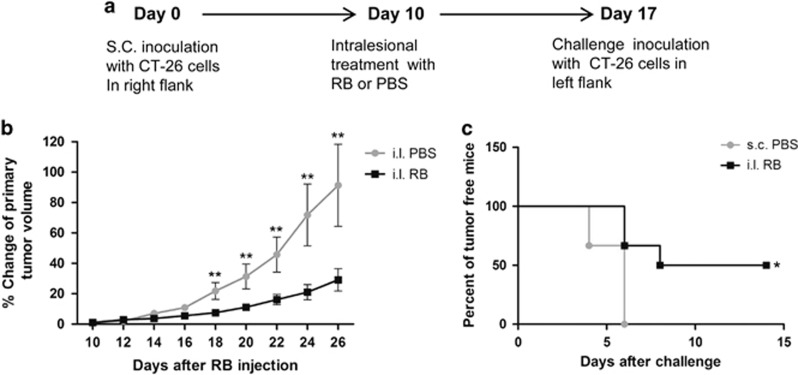
Intralesional injection of RB induces potent antitumor responses in primary CT26 colon cancer tumors and untreated distant CT26 tumors. (**a**) Experimental timeline. (**b**). Intralesional (i.l.) injection of RB resulted in significant growth retardation of primary tumors. (**c**) Intralesional injection of RB in CT26 colon cancer tumors significantly decreased tumor growth of distant CT26 challenge tumors compared with subcutaneous (s.c.) PBS control injected animals (*P<0.05 and **P<0.01)

Considering that generation of antitumor immunity secondary to ICD may be sensitive to specific time points during cell death, and that *in vitro* treated CT26 cells used in a vaccination may only retain DAMPs on the cell surface at certain time points after treatment or during vaccination, we performed *in vivo* treatment of colon cancer cell tumors with intralesional injection of RB followed by tumor rechallenge (Figure 6a). There was significant growth retardation of primary tumors after intralesional injection of RB compared with the tumors injected with PBS (Figure 6b). This finding confirmed the *in vivo* efficacy of intralesional RB-induced tumor inhibition. In animals that received RB treatment, subsequent tumor challenge with CT26 resulted in complete inhibition of tumor formation in 50% of animals and significantly extended the tumor-free time in all animals (Figure 6c).

## Discussion

Compared with melanoma, immunotherapy for solid organ malignancies including colon cancer has met significant challenges. Checkpoint blockade with antibodies including ipilimumab, which have demonstrated impressive antitumor activity in melanoma,^32, 33^ have not resulted in clinical responses in microsatellite-stable advanced colon cancer,^34^ and furthermore, function via generalized immune activation that imparts significant autoimmune side effects. Adoptive cell transfer, also successful in a subset of patients with melanoma, is challenging to use in colon cancer patients because of self-antigens, and a recent trial was closed because of dose-limiting toxicity.^35^ Thus, there remains a gap in our knowledge of how to generate antitumor immune responses in colon cancer and new strategies are required.

The use of intralesional RB treatment in patients with dermal metastases from melanoma demonstrated impressive clinical tumor regressions in injected lesions, and revealed clinical responses in a subset of patients in bystander lesions away from the primary injected tumor.^13, 14^ This combination of findings raised the possibility that RB treatment may induce antitumor immunity, a strategy that is desperately needed in the far more common and lethal disease of colon cancer. Therefore, we were very interested in further evaluating this strategy in colon cancer cells.

To confirm clinical relevance, it was first necessary to establish that RB could impart antitumor responses in established colon cancer tumors. Using the intralesional treatment protocol used in the melanoma phase II clinical trial, it was established that a single RB injection resulted in impressive tumor regressions and complete responses in a subset of colon tumors. Given these findings, we then treated murine and human colon cancer cell lines *in vitro* to confirm cytotoxicity, and demonstrated a titratable effect of RB. With these findings, it was left to establish the heretofore incompletely understood mechanism of RB-induced cell death and its immunogenic therapeutic potential in colon cancer.

Cell cycle analysis revealed G2/M growth arrest; however, only ~15% of the cells displayed hypodiploid DNA content, indicating that apoptosis was not the primary pattern of cell death. When using Annexin V/DAPI staining, a commonly used assay for detecting cell death via apoptosis or necrosis, we noticed that RB induced predominantly necrotic cell death in a dose- and time-dependent manner. As autophagy-deficient cancer cells do not generate therapy-relevant immune responses,^29^ we treated cells in the presence or absence of an autophagy inhibitor, confirming that autophagy had a significant role in RB-mediated colon cancer cell death. That chloroquine, a potent inhibitor of lysosome enzymes, could significantly block RB-induced cell death also remained consistent with evidence supporting the lysosome as a target of RB-mediated cell death,^36, 37, 38^ including evidence that siRNA knockdown of the lysosome enzyme cathepsin B protected melanoma cells from RB-induced cell death.^39^ Thus, our data provided an additional mechanistic connection between lysosome integrity and RB-induced cell death. Given that autophagy appeared to have a role in RB-induced colon cancer cell death, the potential for ICD remained. As opposed to normal apoptosis, which is primarily non-immunogenic, and in many cases tolerogenic, ICD of cancer cells may activate dendritic cells with the generation of a tumor-specific T-cell response.^40^ Therefore, we evaluated RB-treated cells for hallmarks of ICD.

ICD is defined by secretion of DAMPs. CRT, one of the indispensable DAMP proteins, is translocated from the ER lumen to the surface of dying cells where it functions to signal phagocytosis.^41^ Heat-shock protein 90, another critical DAMP, is translocated to the plasma membrane under stress conditions where it stimulates antigen-presenting cell surface receptors and aids in the presentation of tumor antigens on MHC class I. Additionally, ICD is associated with the post-mortem secretion of HMGB1, considered to be a late apoptotic marker whose release into the extracellular space appears crucial for optimal presentation of tumor antigens to dendritic cells via binding to TLR4.^42, 43^ Indeed, our data revealed that RB-treated colon cancer cells expressed the distinct hallmarks of ICD, including enhanced expression of CRT and HSP90 on the cell surface, and the release of HMGB1. Notably, RB induced rapid expression of these ICD markers. The externalization of CRT and HSP90 was initiated as early as 5 and 30 min, respectively, and the peak level of extracellular HMGB1 protein was detected 30 min post-RB treatment. Recently, active secretion of ATP by dying cells during ICD has also been found to have a significant role in activating antigen-presenting cells, and functions as a homing signal for monocytes.^29, 44^ Using a fluorescence probe, we confirmed a marked reduction of intracellular ATP levels within 1 h of RB treatment as a surrogate indicator of ATP release from cells. That the mechanistic studies were performed in CT26 murine colon cancer cells was critical to confirm ICD and then create an immunocompetent syngeneic *in vivo* model for the vaccination studies; however, the ICD phenotype was also confirmed in human colon cancer cell lines. Collectively, our results support that RB induced the hallmarks of ICD.

The operative definition of ICD is generation of a protective immune response to dead cell antigens, suggesting that cells undergoing ICD are immunogenic and can be monitored by vaccination assays.^21, 29, 43, 45^ Therefore, to confirm the ICD phenotype, we vaccinated immunocompetent animals with syngeneic colon cancer cells treated with RB. A key finding was the demonstration that RB-treated CT26 cells could serve as a vaccine against subsequent challenge with the same colon cancer tumor cell in immune competent syngeneic mice. In a prophylactic setting, vaccination with *in vitro* RB-treated CT26 cells resulted in slower tumor growth following inoculation with CT26 colon cancer cells but not syngeneic murine 4T1 breast cancer cells, which suggested a specific antitumor immune response. It should be noted that the antitumor growth impact was most significant in the early stages of tumor growth. Likely, repeated or additional vaccinations would be necessary to generate sustained antitumor immunity. Although effective in testing the operative implications of ICD, a limitation of this strategy was that several key secreted DAMPs (including as ATP and HMGB1) were removed during cell processing for the vaccine. In addition, the optimal expression of multiple DAMPs in the ICD process varies between the molecules; therefore, an optimal single treatment time from which to generate the vaccine is complex. We, therefore, expanded the *in vivo* vaccination study using intralesional RB treatment of established colon cancer cell tumors to allow exposure of the various DAMPs to the immune system. With this strategy, RB administration not only substantially reduced the growth rate of the primary colon cancer cell tumors, as expected, but also prevented subsequent challenge tumor formation in 50% of animals, highlighting the antitumor activity and potential protective immune response generated by vaccination with RB-treated cells that were undergoing ICD.^29^ Using intralesional RB as a treatment strategy is directly translatable into clinical practice and is already being safely performed on multiple human clinical trials that include direct tumor injection into melanoma lesions, breast cancer tumors, soft tissue sarcomas and colorectal liver metastases. Thus, it is practical to consider endoscopic, image-guided, or surgical approaches to direct tumor injections.

In the current study, we have provided several lines of evidence demonstrating that RB exhibited pronounced direct cytotoxicity in colorectal cancer cells both *in vivo* and *in vitro*. Our data also confirmed RB-induced prominent cell growth arrest at the G2/M phase and predominant necrotic cell death that was partially dependent on lysosome function. Furthermore, the findings from the current study identified that RB promoted expression of hallmarks related to ICD in colon cancer cell lines. Vaccination with RB-treated colon cancer cells and intralesional tumor injection resulted in retardation in tumor growth or prevention of subsequent tumor formation following challenge with the same tumor cells. These findings show that RB may serve as an inducer of ICD that contributes to enhanced specific antitumor immunity in colorectal cancer. Additional studies are warranted to elucidate the therapeutic potential of RB-induced ICD.

## Materials and methods

### Cell cultures and reagents

The murine colon carcinoma cell line, CT26, murine mammary carcinoma cell line, 4T1 (gift of Yang-Xin Fu), and three human colon cancer cell lines HCT116, SW480, and HT29 were obtained from American Type Culture Collection (ATCC, Manassas, VA, USA) where each lot was STR profiled. All cell lines were authenticated either by ATCC or IDEXX (4T1), tested for mycoplasma, and used at low passage numbers within 6 months. Cells were grown in the following media: RPMI-1640 (CT26); McCoy's 5A (HCT116); minimum essential medium Eagle (SW480); and Dulbecco's modified Eagle's mMedium (HT29). All media were supplemented with 10% FCS, 2 mM glutamine, and penicillin–streptomycin antibiotic, and the cells were incubated at 37 °C in 5% CO_2_. RB solution of 10% was provided by Provectus Biopharmaceuticals (Knoxville, TN, USA). Annexin V-Biotin Apoptosis Detection Kit, streptavidin-FITC, and streptavidin-APC were purchased from eBioscience (San Diego, CA, USA). Anti-*β*-actin (Santa Cruz Biotechnology, Santa Cruz, CA, USA), anti-CRT (Abcam, Cambridge, MA, USA), anti-HSP90, and anti-GAPDH (Cell Signaling Technology, Danvers, MA, USA) antibodies were purchased and used according to the manufacturer's specifications. DAPI, quinacrin dihydrochloride, and chloroquine diphosphate were purchased from Sigma-Aldrich (St. Louis, MO, USA).

### Cell proliferation and viability assays

A total of 2x10^4^ CT26 or HT29 cells were plated in each well of a 96-well plate. RB was added to achieve concentrations ranging from 25 to 1000 *μ*M. Positive control was achieved by the addition of 50 *μ*M of 5-FU. Cells were treated for 24 h, and cell proliferation was determined using CellTiter96 Aqueous One Solution Cell Proliferation Assay Kit (Promega, Madison, WI, USA) following the manufacturer's instructions with absorbance measured at 490 nm. For Trypan blue viability testing, cells were collected at 1, 2, 4, 24, 48, and 72 h after the addition of RB, diluted 1 : 1 in Trypan blue (0.4% in saline) (Lonza, Walkersville, MD, USA), and the cell number was counted with a hemocytometer under a microscope.

### Cell cycle and cell death analysis

CT26 cells were seeded in a 6-well plate at 3 × 10^5^ cells per well, incubated with increasing concentrations of RB (0, 100, 200, and 300 *μ*M) for 24 h, and processed for cell cycle analysis. For cell death evaluation, both murine and human colon cancer cells were treated with RB for 1, 4, 8, and 16 h, after which both floating and attached cells were collected for further analysis.

Secondary to spectral overlap of RB with propidium iodide, DAPI was used to stain DNA and label dead cells. For cell cycle analysis, harvested cells were fixed with 80% ethanol on ice for 30 min and then incubated with 1 ml of DNA staining solution (1 *μ*g/ml DAPI, 0.1% NP-40 in PBS) at room temperature for 30 min. DNA content was determined with FACS on a CyAn analyzer (Beckman Coulter, Brea, CA, USA) and analyzed with the FlowJo software (Ashland, OR, USA).

Cell death was determined with an Annexin V-Biotin Apoptosis Detection Kit. Briefly, cells were first stained with biotin-conjugated Annexin V in 1x binding buffer at room temperature for 15 min and then incubated with streptavidin-APC for 30 min. After being washed with PBS, cells were resuspended in PBS containing DAPI (0.2 *μ*g/ml) for 5 min followed by FACS analysis. Cell death profile was analyzed with the FlowJo software. Necrotic cells were defined as DAPI+ and DAPI+/Annexin V+, and apoptotic cells were defined as Annexin V+ only.^46^

### Detection of CRT on the cell surface

Cell surface expression of CRT was detected by immunofluorescence staining and FACS analysis. CT26 cells were treated over 5–60 min with 50–300 *μ*M of RB. Human colon cancer cell lines were treated for 4 h. Cells were harvested with trypsin, washed in cold PBS with 3% FBS without fixing, and incubated with rabbit anti-CRT antibody (1 : 250) on ice for 30 min followed by Alexa Fluro-488 labeled goat anti-rabbit IgG (1 : 500 dilution) for 30 min. After washing, cells were resuspended in 0.5 ml PBS containing 0.2 *μ*g/ml DAPI. Equal concentrations of normal rabbit IgG was used as an isotype control. The percent of CRT-positive cells was determined in both alive (DAPI−) and dead (DAPI+) cell populations.

### Determination of intracellular ATP

Cells were treated with or without RB (200 *μ*M) for 30 min, labeled with 5 *μ*M quinacrine for an additional 30 min, and collected and subjected to FACS analysis immediately. MFI of the FL1 channel was determined using the CyAn analyzer and the relative ATP content was expressed as the percentage of MFI relative to untreated cells. This technique avoided RB autofluorescence interference with bioluminescent ATP reaction kits.

### Hmgb1 elisa assay

HMGB1 levels were determined using a Mouse HMGB1 ELISA Kit from Novatein Biosciences (Woburn, MA, USA) according to the protocol. CT26 cells were treated for 30 min with or without RB (200 *μ*M) and 100 *μ*l of each sample was added to a 96-well plate in triplicate. Optical density was read at 450nm using a Bio-Rad Labortories iMark Microplate Reader (Hercules, CA, USA).

### Extraction of cell membrane proteins and western blotting

Cells were harvested by scraping, and cell membrane protein was prepared with a Mem-PER Plus Membrane Protein Extraction Kit (Thermo Fisher Scientific, Waltham, MA, USA). Briefly, cells were washed, resuspended in cell permeabilization buffer, and incubated on ice with frequent vortexing for 15 min. After centrifugation at 16000 *g*, supernatants were collected as cytosolic fractions. The pellets with the membrane fraction were solubilized, centrifuged, and collected. Membrane fractions were confirmed by probing for the plasma membrane marker Na, K-ATPase (no. 3010; Cell Signaling Technology). Whole-cell proteins were extracted with M-Per Mammalian Protein Extraction Reagent (Thermo Fisher Scientific) and supplemented with both protease and phosphatase inhibitor cocktails from EMD Millipore (Billerica, MA, USA). Protein concentrations were determined using a Bradford Protein Assay from Bio-Rad Laboratories (Hercules, CA, USA). Thirty micrograms of each protein sample was resolved by SDS-PAGE and transferred to PVDF membrane, followed by 1 h blocking with 5% non-fat milk in Tris-buffered saline, 0.1% Tween-20 (TBS-T). Blots were probed with primary antibodies overnight at 4 °C, washed, and incubated with the corresponding HRP-labeled secondary antibodies for 1 h at room temperature. Protein signals were revealed by exposing Pheonix Research Products Premium X-Ray Film (Candler, NC, USA) with the blots preincubated with SuperSignal West Pico Chemiluminescent Substrate (Thermo Fisher Scientific). Densitometry analysis of each protein band was performed using the NIH ImageJ software (NIH, Bethesda, MD; http://imagej.nih.gov/ij/).

### *In vivo* colon cancer tumor studies

All mouse experiments were approved by the Institutional Animal Care and Use Committee at the University of Illinois at Chicago and performed in the animal facility (BRL) as per the approved protocol. In all, 18–20 g of female CT26 cell syngeneic Balb/c mice were purchased from Charles River Labs (Wilmington, MA, USA).

#### Direct tumor treatment with intralesional RB

A total of 1 × 10^6^ CT26 cells were subcutaneously inoculated into the flank of a mouse. When tumors were palpable, RB (PV-10) was administered via intralesional injection in an amount equal to half the volume of the tumor (0.5 ml/cm^3^). Tumor growth was measured daily with a caliper and the volume was calculated according to the formula: *V*= 1/2(*d*_long*_d_short_^2^).

#### Vaccination study using *in vitro* RB-treated CT26 cells

CT26 cells were treated *in vitro* with RB (300 *μ*M) for 24 h and the cells were harvested and washed with PBS. A total of 1 × 10^6^ RB-treated cells in 100 *μ*l PBS were injected subcutaneously into the upper right flank of each mouse, on days −14 and −7, whereas control mice received 100 *μ*l injections of PBS. One week after the second vaccination (day 0), 1 × 10^6^ untreated CT26 cells and 1 × 10^6^ 4T1 murine breast cancer cells were inoculated into the lower right flank and upper left flank, respectively. Mice were killed 2 weeks after the challenge injection and the tumors were excised in their entirety.

#### Vaccination study using *in vivo* RB-treated tumors

Mice received subcutaneous inoculation of CT26 cells (1 × 10^6^/100 *μ*l) or PBS injection (100 *μ*l) in the right flank. When tumors were palpable in CT26-injected mice, these animals were randomized into two groups (six mice per group), and tumors were injected with 50 *μ*l of RB solution or PBS. At 1 week after intralesional injection, all mice were challenged by subcutaneous injection of 1 × 10^6^ CT26 cells in the left flank. Tumor growth was monitored every 2 days. Two weeks after challenge injection with CT26 cells, all mice were killed and tumors were excised. Animals that reached humane end points before 2 weeks were killed.

### Statistics

All quantitative data are expressed as mean±S.E. and differences between groups were evaluated with a two-tailed *T*-test. Significance was defined as *P*<0.05. Comparisons in tumor-free time between groups was determined with the log-rank test.

## References

[bib1] Siegel RL, Miller KD, Jemal A. Cancer statistics, 2015. CA Cancer J Clin 2015; 65: 5–29. 25559415 10.3322/caac.21254

[bib2] Galon J, Costes A, Sanchez-Cabo F, Kirilovsky A, Mlecnik B, Lagorce-Pages C et al. Type, density, and location of immune cells within human colorectal tumors predict clinical outcome. Science 2006; 313: 1960–1964. 17008531 10.1126/science.1129139

[bib3] Pagès F, Berger A, Camus M, Sanchez-Cabo F, Costes A, Molidor R et al. Effector memory T cells, early metastasis, and survival in colorectal cancer. N Engl J Med 2005; 353: 2654–2666. 16371631 10.1056/NEJMoa051424

[bib4] Katz SC, Bamboat ZM, Maker AV, Shia J, Pillarisetty VG, Yopp AC et al. Regulatory T cell infiltration predicts outcome following resection of colorectal cancer liver metastases. Ann Surg Oncol 2013; 20: 946–955. 23010736 10.1245/s10434-012-2668-9 PMC3740360

[bib5] Maker AV, Ito H, Mo Q, Weisenberg E, Qin LX, Turcotte S et al. Genetic evidence that intratumoral T-cell proliferation and activation are associated with recurrence and survival in patients with resected colorectal liver metastases. Cancer immunology research 2015; 3: 380–388. 25600439 10.1158/2326-6066.CIR-14-0212 PMC4390462

[bib6] Maker AV. Precise identification of immunotherapeutic targets for solid malignancies using clues within the tumor microenvironment—evidence to turn on the LIGHT. Oncoimmunology 2016; 5: e1069937. 26942091 10.1080/2162402X.2015.1069937 PMC4760328

[bib7] Epstein NND, Hsiung GD. Rose bengal test for liver function. Can Med Assoc J 1927; 17: 878–880. 20316443 PMC407475

[bib8] Forster HWJr. Rose bengal test in diagnosis of deficient tear formation. AMA Arch Ophthalmol 1951; 45: 419–424. 14818497 10.1001/archopht.1951.01700010429008

[bib9] O'Day K. Bengal rose as an aid in the diagnosis of kerato-conjunctivitis sicca (Sjogren's syndrome). Med J Aust 1951; 2: 708–709. 14899116

[bib10] Kubin R, Grodsky G, Carbone J. Investigation of rose bengal conjugation. Proc Soc Exp Biol Med 1960; 140: 650–653. 10.3181/00379727-104-25939 13754777

[bib11] Marsh RJ, Fraunfelder FT, McGill JI. Herpetic corneal epithelial disease. Arch Ophthalmol 1976; 94: 1899–1902. 62568 10.1001/archopht.1976.03910040609004

[bib12] Pirotte J. Suitability criteria for compartmental analysis of the plasma clearance curve of exogenous cholephils. ^131^I rose bengal fulfils these criteria. Biomedicine 1979; 30: 211–215. 534674

[bib13] Ross MI. Intralesional therapy with PV-10 (rose bengal) for in-transit melanoma. J Surg Oncol 2014; 109: 314–319. 24510477 10.1002/jso.23554

[bib14] Thompson JF, Agarwala SS, Smithers BM, Ross MI, Scoggins CR, Coventry BJ et al. Phase 2 study of intralesional PV-10 in refractory metastatic melanoma. Ann Surg Oncol 2015; 22: 2135–2142. 25348780 10.1245/s10434-014-4169-5 PMC4458269

[bib15] Thompson JF, Hersey P, Wachter E. Chemoablation of metastatic melanoma using intralesional rose bengal. Melanoma Res 2008; 18: 405–411. 18830132 10.1097/CMR.0b013e32831328c7

[bib16] Mousavi HZX, Gillespie S, Wachter E, Hersey P. Rose bengal induces dual modes of cell death in melanoma cells and has clinical activity against melanoma. Melanoma Res 2006; 16: S8.

[bib17] Maker AV, Prabhakar B, Pardiwala K. The potential of intralesional rose bengal to stimulate T-cell mediated anti-tumor responses. J Clin Cell Immunol 2015; 6: 1–6. 10.4172/2155-9899.1000343 PMC4662376 26618054

[bib18] Koevary SB. Selective toxicity of rose bengal to ovarian cancer cells *in vitro*. Int J Physiol Pathophysiol Pharmacol 2012; 4: 99–107. 22837809 PMC3403562

[bib19] Zamani Taghizadeh Rabe S, Mousavi SH, Tabasi N, Rastin M, Zamani Taghizadeh Rabe S, Siadat Z et al. Rose bengal suppresses gastric cancer cell proliferation via apoptosis and inhibits nitric oxide formation in macrophages. J Immunotoxicol 2014; 11: 367–375. 24575814 10.3109/1547691X.2013.853715

[bib20] Wachter E, Dees C, Harkins J, Fisher W, Scott T. Imaging photosensitizer distribution and pharmacology using multiphoton microscopy. Proc SPIE 2002; 4620: 112–118.

[bib21] Green DR, Ferguson T, Zitvogel L, Kroemer G. Immunogenic and tolerogenic cell death. Nat Rev Immunol 2009; 9: 353–363. 19365408 10.1038/nri2545 PMC2818721

[bib22] Bodin P, Burnstock G. Evidence that release of adenosine triphosphate from endothelial cells during increased shear stress is vesicular. J Cardiovasc Pharmacol 2001; 38: 900–908. 11707694 10.1097/00005344-200112000-00012

[bib23] Garg AD, Dudek AM, Agostinis P. Cancer immunogenicity, danger signals, and DAMPs: what, when, and how? Biofactors 2013; 39: 355–367. 23900966 10.1002/biof.1125

[bib24] Garg AD, Galluzzi L, Apetoh L, Baert T, Birge RB, Bravo-San Pedro JM et al. Molecular and translational classifications of DAMPs in immunogenic cell death. Front Immunol 2015; 6: 588. 26635802 10.3389/fimmu.2015.00588 PMC4653610

[bib25] Garg AD, Martin S, Golab J, Agostinis P. Danger signalling during cancer cell death: origins, plasticity and regulation. Cell Death Differ 2014; 21: 26–38. 23686135 10.1038/cdd.2013.48 PMC3858605

[bib26] Hou W, Zhang Q, Yan Z, Chen R, Zeh Iii HJ, Kang R et al. Strange attractors: DAMPs and autophagy link tumor cell death and immunity. Cell Death Dis 2013; 4: e966. 24336086 10.1038/cddis.2013.493 PMC3877563

[bib27] Panzarini E, Inguscio V, Fimia GM, Dini L. Rose bengal acetate photodynamic therapy (RBAc-PDT) induces exposure and release of damage-associated molecular patterns (DAMPs) in human HeLa cells. PLoS One 2014; 9: e105778. 25140900 10.1371/journal.pone.0105778 PMC4139382

[bib28] Tan CY, Neuhaus SJ. Novel use of rose bengal (PV-10) in two cases of refractory scalp sarcoma. ANZ J Surg 2013; 83: 93. 10.1111/ans.12033 23350981

[bib29] Martins I, Wang Y, Michaud M, Ma Y, Sukkurwala AQ, Shen S et al. Molecular mechanisms of ATP secretion during immunogenic cell death. Cell Death Differ 2014; 21: 79–91. 23852373 10.1038/cdd.2013.75 PMC3857631

[bib30] Qin JZ, Upadhyay V, Prabhakar B, Maker AV. Shedding LIGHT (TNFSF14) on the tumor microenvironment of colorectal cancer liver metastases. J Transl Med 2013; 11: 70. 23514280 10.1186/1479-5876-11-70 PMC3623860

[bib31] Krysko DV, Vanden Berghe T, D'Herde K, Vandenabeele P. Apoptosis and necrosis: detection, discrimination and phagocytosis. Methods 2008; 44: 205–221. 18314051 10.1016/j.ymeth.2007.12.001

[bib32] Maker AV, Attia P, Rosenberg SA. Analysis of the cellular mechanism of antitumor responses and autoimmunity in patients treated with CTLA-4 blockade. J Immunol 2005; 175: 7746–7754. 16301685 10.4049/jimmunol.175.11.7746 PMC1473972

[bib33] Maker AV, Phan GQ, Attia P, Yang JC, Sherry RM, Topalian SL et al. Tumor regression and autoimmunity in patients treated with cytotoxic T lymphocyte-associated antigen 4 blockade and interleukin 2: a phase I/II study. Ann Surg Oncol 2005; 12: 1005–1016. 16283570 10.1245/ASO.2005.03.536 PMC1473970

[bib34] Chung KY, Gore I, Fong L, Venook A, Beck SB, Dorazio P et al. Phase II study of the anti-cytotoxic T-lymphocyte-associated antigen 4 monoclonal antibody, tremelimumab, in patients with refractory metastatic colorectal cancer. J Clin Oncol 2010; 28: 3485–3490. 20498386 10.1200/JCO.2010.28.3994

[bib35] Parkhurst MR, Yang JC, Langan RC, Dudley ME, Nathan DA, Feldman SA et al. T cells targeting carcinoembryonic antigen can mediate regression of metastatic colorectal cancer but induce severe transient colitis. Mol Ther 2010; 19: 620–626. 21157437 10.1038/mt.2010.272 PMC3048186

[bib36] Mousavi SH, Tavakkol-Afshari J, Brook A, Jafari-Anarkooli I. Direct toxicity of rose bengal in MCF-7 cell line: role of apoptosis. Food Chem Toxicol 2009; 47: 855–859. 19271285 10.1016/j.fct.2009.01.018

[bib37] Wachter E, Dees C, Harkins J, Scott T, Petersen M, Rush RE et al. Topical rose bengal: pre-clinical evaluation of pharmacokinetics and safety. Lasers Surg Med 2003; 32: 101–110. 12561042 10.1002/lsm.10138

[bib38] Wachter E, Dees C, Harkins J, Fisher W, Scott T. Functional imaging of photosensitizers using multiphoton microscopy. Proc SPIE 2002; 4620: 143–147.

[bib39] Toomey P, Kodumudi K, Weber A, Kuhn L, Moore E, Sarnaik AA et al. Intralesional injection of rose bengal induces a systemic tumor-specific immune response in murine models of melanoma and breast cancer. PLoS One 2013; 8: e68561. 23874673 10.1371/journal.pone.0068561 PMC3714270

[bib40] Spisek R, Dhodapkar MV. Towards a better way to die with chemotherapy: role of heat shock protein exposure on dying tumor cells. Cell Cycle 2007; 6: 1962–1965. 17721082 10.4161/cc.6.16.4601

[bib41] Obeid M, Tesniere A, Ghiringhelli F, Fimia GM, Apetoh L, Perfettini JL et al. Calreticulin exposure dictates the immunogenicity of cancer cell death. Nat Med 2007; 13: 54–61. 17187072 10.1038/nm1523

[bib42] Vacchelli E, Galluzzi L, Rousseau V, Rigoni A, Tesniere A, Delahaye N et al. Loss-of-function alleles of P2RX7 and TLR4 fail to affect the response to chemotherapy in non-small cell lung cancer. Oncoimmunology 2012; 1: 271–278. 22737602 10.4161/onci.18684 PMC3382853

[bib43] Kroemer G, Galluzzi L, Kepp O, Zitvogel L. Immunogenic cell death in cancer therapy. Annu Rev Immunol 2013; 31: 51–72. 23157435 10.1146/annurev-immunol-032712-100008

[bib44] Garg AD, Krysko DV, Verfaillie T, Kaczmarek A, Ferreira GB, Marysael T et al. A novel pathway combining calreticulin exposure and ATP secretion in immunogenic cancer cell death. EMBO J 2012; 31: 1062–1079. 22252128 10.1038/emboj.2011.497 PMC3298003

[bib45] Galluzzi L, Kepp O, Kroemer G. Mitochondria: master regulators of danger signalling. Nat Rev Mol Cell Biol 2012; 13: 780–788. 23175281 10.1038/nrm3479

[bib46] Vanden Berghe T, Grootjans S, Goossens V, Dondelinger Y, Krysko DV, Takahashi N et al. Determination of apoptotic and necrotic cell death *in vitro* and *in vivo*. Methods 2013; 61: 117–129. 23473780 10.1016/j.ymeth.2013.02.011

