# Supplementary material for: Colon cancer cell treatment with rose bengal generates a protective immune response via immunogenic cell death
Source: Cell Death Dis. 2017 Feb 2;8(2):e2584–. doi: 10.1038/cddis.2016.473 (PMC5386459; doi:10.1038/cddis.2016.473)
Supplement: Supplementary Information [file cddis2016473x1.docx]

**Supplemental Figures**


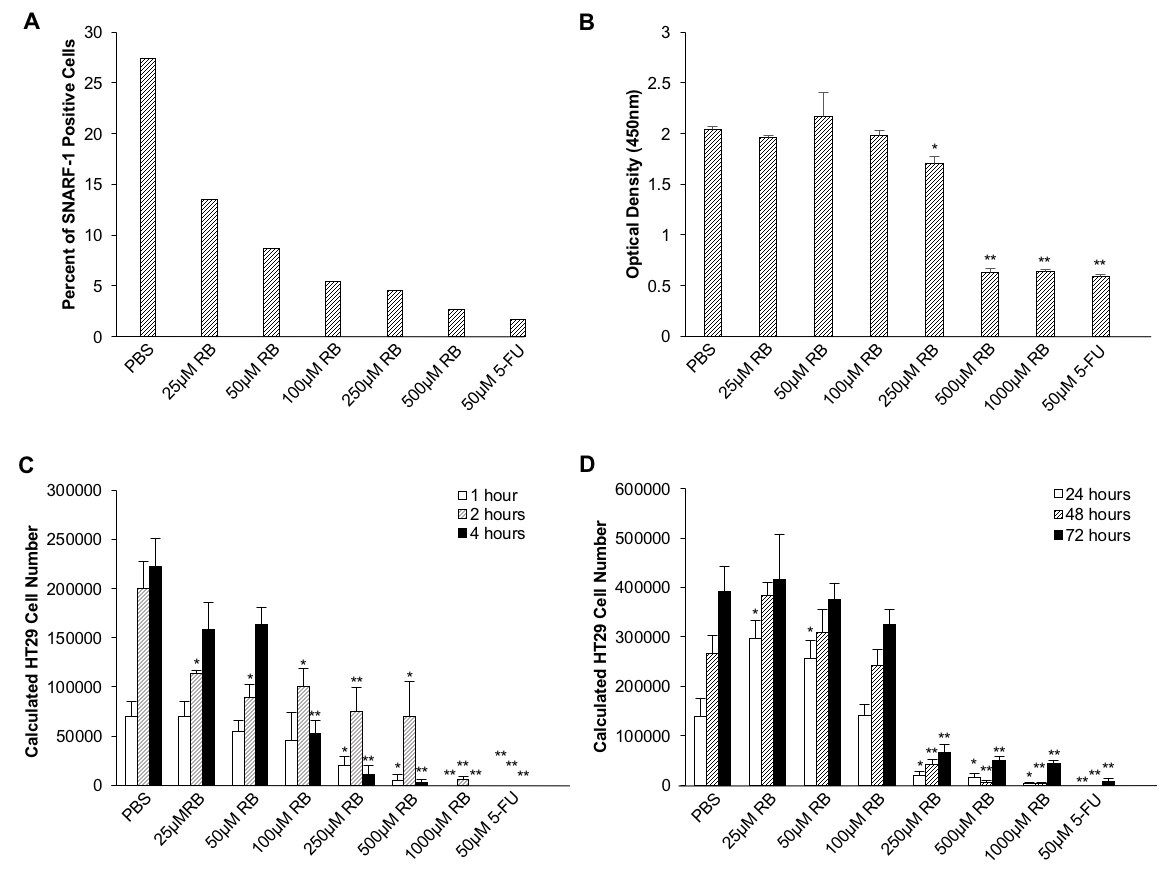


*Supplemental Figure 1: Rose Bengal induces cell death in HT29 cells*

**A.** Intracellular pH increased with RB treatment as determined with a flow cytometry based assessment of SNARF-1 expression upon RB treatment. 5-Fluorouracil, a known cytotoxic chemotherapeutic agent for colon cancer, was used as a positive control. **B.** MTS assay performed 24 hours after RB exposure revealed increased cell death with increasing RB concentrations, similar to treatment with 5-FU. Trypan Blue exclusion evaluated cell viability at 1, 2, and 4 hrs (**C**) and 24, 48, and 72hrs (**D**) demonstrating significant RB-induced cell death upon exposure. (* p < 0.05; ** p < 0.01)


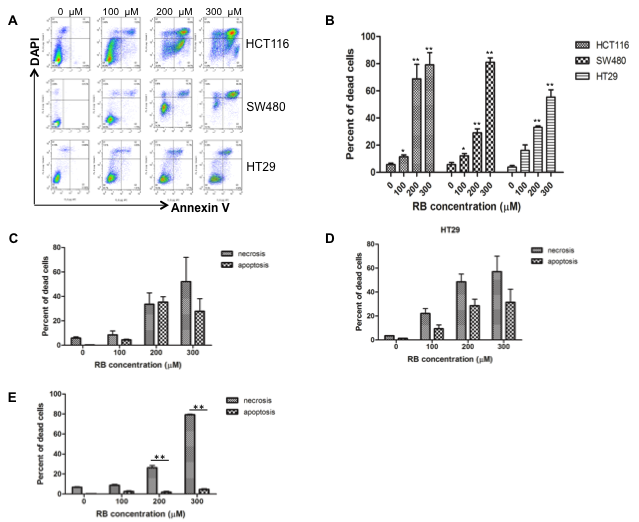


*Supplemental Figure 2: Mechanism of Rose Bengal-induced cell death*

**A.** Representative flow cytometry analysis of RB-induced cell death in human colon cancer cell lines HCT116, SW480, & HT29 with increasing doses of RB. **B**. Pooled data from three experiments revealed increased cell death in all cell lines with increasing doses of RB treatment. **C.** RB-treated HT116 cells show increased necrosis and apoptosis. **D.** RB-treated HT29 cells show increased necrosis and apoptosis. **E.** RB-treated SW480 cells show predominantly necrosis.


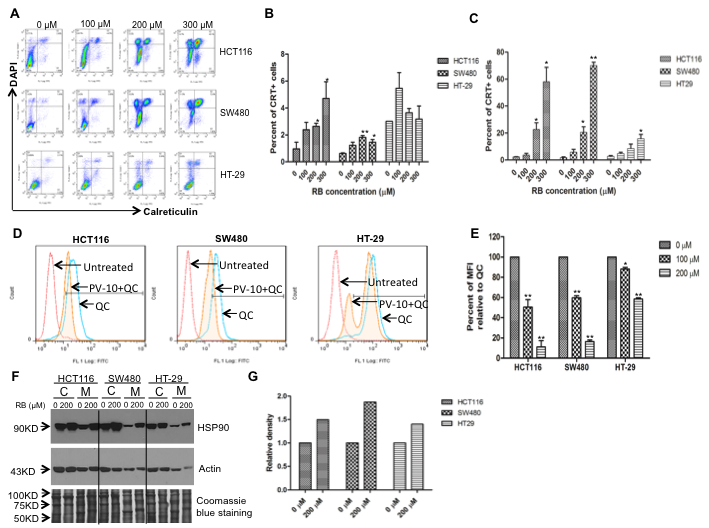


*Supplemental Figure 3: Rose Bengal induces release and/or increases expression of DAMPs in human cell lines*

**A.** Representative FACS analysis of RB-treated HCT116, SW480, & HT29 reveals increased expression of CRT with increasing doses of drug. **B.** RB induced a significant increase in surface expression of CRT in viable cells (**(**DAPI-/CRT+) in HCT116 and SW480 cells, and only trended to increase in HT29 cells. **C.** RB induced a significant increase in surface expression of CRT in dead cells **(**DAPI+/CRT+) in HCT116, SW480, and HT29 cells. **D & E.** Treatment with RB decreased intracellular ATP levels in HCT116, SW480, & HT29. A representative histogram (D) and summary of three separate experiments (E) demonstrate a decrease in intracellular ATP levels upon treatment with RB. **F.** Western blot reveals HSP90 levels in cytosol (C) and the cell membrane (M) after treatment with 200 μM RB. **G.** Treatment with RB increased HSP90 expression on the cell membrane of HCT116, SW480, and HT29 cells.

(* p < 0.05; ** p < 0.01)
